# Fitness Cost of Fluoroquinolone Resistance in Clinical Isolates of *Pseudomonas aeruginosa* Differs by Type III Secretion Genotype

**DOI:** 10.3389/fmicb.2016.01591

**Published:** 2016-10-04

**Authors:** Melissa Agnello, Steven E. Finkel, Annie Wong-Beringer

**Affiliations:** ^1^School of Pharmacy, University of Southern CaliforniaLos Angeles, CA, USA; ^2^Molecular and Computational Biology Section, Department of Biological Sciences, University of Southern CaliforniaLos Angeles, CA, USA

**Keywords:** *Pseudomonas aeruginosa*, fluoroquinolone resistance, type III secretion, fitness, virulence

## Abstract

Fluoroquinolone (FQ) resistance is highly prevalent among clinical strains of *Pseudomonas aeruginosa*, limiting treatment options. We have reported previously that highly virulent strains containing the *exoU* gene of the type III secretion system are more likely to be FQ-resistant than strains containing the *exoS* gene, as well as more likely to acquire resistance-conferring mutations in *gyrA/B* and *parC/E*. We hypothesize that FQ-resistance imposes a lower fitness cost on *exoU* compared to *exoS* strains, thus allowing for better adaptation to the FQ-rich clinical environment. We created isogenic mutants containing a common FQ-resistance conferring point mutation in *parC* from three *exoU* to three *exoS* clinical isolates and tested fitness *in vitro* using head-to-head competition assays. The mutation differentially affected fitness in the *exoU* and *exoS* strains tested. While the addition of the *parC* mutation dramatically increased fitness in one of the *exoU* strains leaving the other two unaffected, all three *exoS* strains displayed a general decrease in fitness. In addition, we found that *exoU* strains may be able to compensate for the fitness costs associated with the mutation through better regulation of supercoiling compared to the *exoS* strains. These results may provide a biological explanation for the observed predominance of the virulent *exoU* genotype in FQ-resistant clinical subpopulations and represent the first investigation into potential differences in fitness costs of FQ-resistance that are linked to the virulence genotype of *P. aeruginosa*. Understanding the fitness costs of antibiotic resistance and possibilities of compensation for these costs is essential for the rational development of strategies to combat the problem of antibiotic resistance.

## Introduction

*Pseudomonas aeruginosa* is a Gram-negative pathogen that is the leading cause of nosocomial pneumonia (Restrepo and Anzueto, 2009; Quartin et al., 2013). Resistance to the fluoroquinolone (FQ) antibiotics has risen dramatically due to the widespread prescribing of this class of drug, limiting treatment options for *P. aeruginosa* infections (Linder et al., 2005). *P. aeruginosa* acquires resistance to the FQs through mutations in genes regulating the expression of efflux pumps and through point mutations in target site genes. The target enzymes of the FQs are the type II topoisomerases, GyrA/B and ParC/E (Hooper, 2001). Resistance-conferring mutations in these genes, known as target site mutations, have been well described in *P. aeruginosa* (Nakano et al., 1997; Mouneimné et al., 1999; Higgins et al., 2003).

*Pseudomonas aeruginosa* has the ability to cause a variety of severe infections due to its many virulence factors. Specifically, *P. aeruginosa* utilizes the type III secretion system (TTSS) during acute infections to evade phagocytosis, invade host cells, and cause cell death (Veesenmeyer et al., 2009). The TTSS consists of a molecular syringe-like apparatus that extends through the inner and outer membranes and directly contacts the host cell. This allows effector toxins (ExoU, ExoS, ExoY, and/or ExoT) to be directly injected into the cytoplasm of host cells. Although residing at entirely separate loci, the genes encoding the toxins ExoU and ExoS are mutually exclusive in most strains, with the *exoS* genotype accounting for about 70% of clinical and environmental strains (Feltman et al., 2001; Garey et al., 2008). While less prevalent in general, *exoU* strains are more virulent than *exoS* strains, as has been shown in animal models of acute pneumonia (Shaver and Hauser, 2004). Importantly, infection with these strains leads to poor outcomes in patients with ventilator-associated pneumonia (Roy-Burman et al., 2001; El Solh et al., 2008) as well as increased persistence and severity of disease (Schulert et al., 2003).

Alarmingly, clinical studies have shown a correlation between FQ-resistance and virulence. We have previously reported that patients infected with FQ-resistant strains of *P. aeruginosa* had threefold higher mortality or prolonged illness by an additional 5 days compared to those infected with FQ-susceptible strains (Hsu et al., 2005). In addition, clinical isolates with the more virulent *exoU* genotype were shown to more likely be FQ-resistant than *exoS* strains (Wong-Beringer et al., 2008; Agnello and Wong-Beringer, 2012). Others have reported similar results in isolates from various infection sites (Zhang et al., 2014; Peña et al., 2015). Furthermore, in a separate study, we found that the combined traits of FQ-resistance and *exoU* genotype among respiratory isolates of *P. aeruginosa* were significantly associated with the development of pneumonia rather than bronchitis or colonization (Sullivan et al., 2014), suggesting that resistance and virulence traits may be linked, negatively impacting disease severity.

In a large study of 270 clinical isolates, we found that significantly more *exoU* strains were FQ-resistant, compared to *exoS* strains (63% vs. 49%, *p* = 0.03). Sequencing of the FQ target site genes *gyrA/B* and *parC/E* revealed that *exoU* strains were more likely than *exoS* strains to acquire two or more resistance-conferring target site mutations (Agnello and Wong-Beringer, 2012). Specifically, we found that while FQ-resistant *exoU* and *exoS* strains were similarly likely to possess a mutation in *gyrA*, *exoU* isolates were more likely to also have acquired an additional mutation in *parC*, resulting in greatly increased levels of resistance. While there is mounting evidence for the co-selection of resistance and virulence traits among pathogenic bacteria (Beceiro et al., 2013), the biological mechanisms underlying these observations are not well understood. Since the identification of the ExoU and ExoS toxins of the *P. aeruginosa* type III secretion system, many studies have investigated the roles of each toxin during infection (Allewelt et al., 2000; Schulert et al., 2003; Shaver and Hauser, 2004, 2006) while others have described the association of *exoU* strains with increased FQ resistance (Lakkis and Fleiszig, 2001; Maatallah et al., 2011) supporting our own observations. The aim of the current study was to gain insights into the biological basis underlying the differential development of resistance in *exoU* vs. *exoS* strains, which previous studies have not explored. We hypothesize that the greater propensity of *exoU* strains to acquire multiple target site mutations compared to *exoS* strains reflects a difference in the fitness costs associated with FQ-resistance mutations, favoring the strains with the *exoU* genetic background.

A recent meta-analysis of studies conducted on fitness costs of resistance to a variety of antibiotic classes in different organisms showed that most point mutations are generally costly (Melnyk et al., 2015). However, there is great variability depending on organism, drug, and mechanism of resistance. Furthermore, fitness costs can be mitigated through the accumulation of compensatory mutations that restore fitness to wild-type levels without loss of resistance (Andersson and Hughes, 2010). FQ-conferring mutations have been shown to impose variable fitness costs, depending on strain background and specific combinations of mutations (Komp Lindgren et al., 2005; Luo et al., 2005; Marcusson et al., 2009; Machuca et al., 2014; Wasels et al., 2015).

In the current study, we compared the fitness effects of a target site mutation in *parC* using isogenic mutants created from clinical *exoU* and *exoS* isolates. We investigated the fitness effects of this mutation using *in vitro* head-to-head competition experiments. Changes in supercoiling level and mutation frequency between mutants and parent strains were also assessed to explore potential mechanisms for the difference in fitness observed.

Our results suggest that the FQ resistance-conferring point mutation in *parC* studied here may confer less of a fitness cost to *exoU* strains than to *exoS* strains, with *exoU* strains showing evidence of compensation for the fitness costs. These results provide a potential biological explanation for the observed predominance of *exoU* strains in the clinical FQ-resistant population. The co-selection of FQ-resistance in highly virulent *exoU* strains will likely have a negative impact on patient outcomes, which underscores the importance to gain a better understanding of the biological basis for this observation.

## Materials and Methods

### Ethics Statement

The institutional review boards at both Huntington Hospital and the University of Southern California have approved this study. Informed consent was waived from all participants since bacteria cultures were saved as part of a longitudinal epidemiological surveillance study. All data from respiratory cultures were analyzed anonymously to protect patient privacy.

### Bacterial Strains and Culture Conditions

Strains of *P. aeruginosa* used in this study (**Table 1**) were selected from a collection of isolates obtained from the respiratory tract of hospitalized patients at Huntington Hospital, Pasadena, CA, USA from 2005 to 2009 and were stored in cryovials at -80°C in 30% glycerol. All strains had been previously characterized for: the presence of the *exoU* or *exoS* gene, resistance to levofloxacin, presence of mutations in the quinolone-resistance determining regions of *gyrA/B* and *parC/E*, and clonal relatedness by RAPD PCR, as described in Agnello and Wong-Beringer (2012). Strains were routinely grown as 5 ml cultures in Luria-Bertani (LB) broth in 12 ml polypropylene culture tubes at 37°C and shaking at 250 rpm for liquid culture, or grown on *Pseudomonas* Isolation Agar (PIA) plates at 37°C. Strains were routinely sub-cultured twice after inoculation from the frozen stock by 1:100 dilution (vol:vol) before use in any experiment.

**Table 1:** Characteristics of Strains.

	Name	LVX^a^ MIC (μg/ml)	LVX + EPI^b^ MIC (μg/ml)	Amino Acid Change	Doubling Time, h (-/+ SEM)^c^
*exoU*	U-37	16	0.5	GyrA: T83I	2.0 (1.7–2.4)
	U-37PC^∗^	16	0.5	GyrA: T83I ParC: S87L	1.7 (1.5–1.9)
	U-91	2	2	GyrA: T83I	3.9 (3.78–3.93)
	U-91PC^∗^	16	2	GyrA: T83I ParC: S87L	3.9 (3.87–3.96)
	U-92	16	0.5	GyrA: T83I	2.5 (2.3–2.7)
	U-92PC^∗^	16	0.5	GyrA: T83I ParC: S87L	2.8 (2.6–3.0)
*exoS*	S-139	4	0.5	GyrA: T83I	3.4 (3.3–3.6)
	S-139PC^∗^	32	0.25	GyrA: T83I ParC: S87L	3.1 (2.9–3.3)
	S-247	16	1	GyrA: T83I	2.5 (2.4–2.6)
	S-247PC^∗^	16	8	GyrA: T83I ParC: S87L	2.5 (2.4–2.7)
	S-215	16	1	GyrA: T83I	2.4 (2.2–2.7)
	S-215PC^∗^	16	1	GyrA: T83I ParC: S87L	2.3 (2.2–2.5)

*Mutants with Ser87Leu substitution in ParC denoted PC^∗^. ^a^LVX, Levofloxacin; ^b^EPI, efflux pump inhibitor; ^c^Doubling time during exponential phase.*

### Susceptibility Testing and Sequencing

Susceptibility testing to levofloxacin and rifampicin was performed by broth microdilution in twofold dilutions at concentrations ranging from 0.25 to 128 μg/ml according to guidelines recommended by CLSI (Clinical and Laboratory Standards Institute, 2007). In order to assess the involvement of the multidrug Mex efflux pumps to resistance, MIC of levofloxacin was also measured with the addition of an efflux pump inhibitor (EPI, MC-0228; Sigma) at 20 μg/ml (Kriengkauykiat et al., 2005).

For sequencing, genomic DNA was extracted and purified from isolates using the DNeasy Mini Kit (Qiagen). The quinolone-resistance determining regions of target genes *gyrA, gyrB, parC*, and *parE* were amplified by PCR using previously published primers and conditions and sequenced to identify mutations compared to wild-type strain PAO1 (**Table 2**) (Jalal and Wretlind, 1998; Lee et al., 2005; Winsor et al., 2011).

**Table 2:** Genetic elements used.

	Sequence	Reference
**Primer name PCR**		
*glmS*-up	CTGTGCGACTGCTGGAGCTGA	Choi and Schweizer, 2006
*glmS*-down	GCACATCGGCGACGTGCTCTC	Choi and Schweizer, 2006
Tn7-R	CACAGCATAACTGGACTGATTTC	Choi and Schweizer, 2006
Tn7-L	ATTAGCTTACGACGCTACACC	Choi and Schweizer, 2006
**Oligo for recombination**		
PC^∗^	TGCTCGGCAAGTTCCACCCGCACGGCGACTTGGCCTGCTACGAGGCCATGGTGCTGATGG	Agnello and Wong-Beringer, 2012

**Plasmid**	**Function**	**References**

pTNS3	Helper plasmid, encodes Tn-7 transposition pathway	Choi et al., 2005
pUC18T-mini-Tn7T-Gm-eyfp	Delivery plasmid for Gm^R^ marker and YFP	Choi and Schweizer, 2006
pUC18T-mini-Tn7T-Gm-ecfp	Delivery plasmid for Gm^R^ marker and CFP	Choi and Schweizer, 2006
TOP10/mini-Tn7-*PA0614* promoter-Gm-luxCDABE	Delivery plasmid for Gm^R^ marker and *lux* operon	Choi and Schweizer, 2006

### Creation of Target Site Mutants

Since most FQ-resistant isolates have *gyrA* mutations, three *exoU* and three *exoS* isolates with a pre-existing *gyrA* FQ-resistance mutation (Thr83→Ile substitution) were selected for mutagenesis. A site-directed point mutation in the *parC* gene leading to the amino acid substitution Ser87→Leu was introduced via electroporation of an oligonucleotide in order to create isogenic mutants, using the technique of oligonucleotide recombination as described previously (Swingle et al., 2010; Agnello and Wong-Beringer, 2014). Briefly, a single-stranded oligonucleotide 60 bases in length was designed using the *parC* gene sequence of reference strain PAO1 (Winsor et al., 2011) (**Table 2**). The oligo is identical to the PAO1 sequence from nucleotides 230 to 289, save for the point mutation TCG→TTG at location 31 of the oligo, corresponding to nucleotide 260 in the *parC* gene. This point mutation, which is the most common *parC* mutation observed among fluoroquinolone-resistant clinical strains, gives rise to the Ser87→Leu amino acid change in the ParC protein.

Strains were made electro-competent by several washes with 300 mM sucrose solution as described in Choi et al. (2006) and transformed by electroporation at 2.5 kV in a 0.2 cm cuvette using a Micropulser (Bio-Rad, Hercules, CA, USA). SOC medium (1 ml) was immediately added and the cells were outgrown overnight at 37°C on PIA plus levofloxacin at concentrations two and fourfold above the original minimum inhibitory concentration (MIC) of the isolates for selection. Single colonies were collected and the *parC* gene was sequenced as described above to confirm that recombination had occurred.

### Growth Rate Measurements

Independent overnight cultures were diluted to OD_600_ ~0.1 and grown at 37°C with shaking in 50 ml LB broth in a 100 ml flask. An aliquot of the culture (150 μl) was sampled every 30 min for 8 h and turbidity was measured at OD_600_ using a Tecan Sunrise microplate reader (Tecan Group Ltd., Switzerland). Results were reported as an average of at least three independent experiments in terms of doubling time per hour.

### Insertion of Fluorescent Tag

Mini-Tn7 vectors developed and generously shared with us by Dr. Herbert Schweizer (Choi et al., 2005) were utilized to insert cassettes containing the genes encoding YFP or CFP as well as a cassette containing the *lux* operon into strains used for competition experiments and supercoiling experiments, respectively. The cassette inserts into the *P. aeruginosa* genome at a single location (*att*Tn7 site downstream of the *glmS* gene) and contains the gene for the fluorescent protein under the control of a constitutive promoter or the *lux* operon under the control of a supercoiling-sensitive promoter (Moir et al., 2007), as well as a gentamicin-resistance marker for selection. The delivery plasmid was co-electroporated with a helper plasmid (pTNS3) encoding the necessary transposase function for insertion. The electroporation protocol used for transformation is described in detail in Choi and Schweizer (2006); briefly, strains were made electro-competent through a series of washes with 300 mM sucrose, subjected to electroporation as described above, and plated on LB plates containing 30 μg/ml gentamicin for selection. To confirm insertion had occurred, PCR was performed to amplify the insertion region using primers listed in **Table 2** and protocol as previously described (Choi and Schweizer, 2006) (data not shown).

### *In vitro* Competition Assays

To investigate the effects of the *parC* mutation (PC^∗^) on fitness, mutants were directly competed against isogenic parent strains in co-culture. Strains were tagged with either CFP or YFP for differentiation as described above. PC^∗^ mutant strains were initially tagged with CFP, and competed against the respective isogenic parent strain tagged with YFP. To confirm neutrality of the tags, experiments were also performed with YFP-mutants and CFP-parents.

Strains were grown overnight in LB, and 10^5^ CFU of each strain was co-inoculated into 10 ml LB in a 50 ml flask, and grown with shaking at 37°C. Every 24 h, 10 μl of the culture was transferred to a new flask containing 10 ml fresh LB, and a sample of the culture was serially diluted then plated on PIA for CFU enumeration. Colonies of each color were counted using a fluorescent wide-field microscope (Zeiss Axio Zoom.V16). Experiment duration ranged from 4 to 7 days. The ratio of the number of CFUs of the mutant-to-parent strain on each day of the experiment was calculated and used to determine the relative fitness of the mutant. Experiments were repeated a minimum of five times. Assays were also performed with the addition of levofloxacin equal to 1/8 the MIC (2 or 0.5 μg/ml). At day 7 of each competition experiment, colonies were selected from plates, inoculated into LB, grown overnight, and stored at -80°C in 30% glycerol until ready for use in subsequent experiments. These were described as ‘aged’ strains. Aged strains were subjected to a secondary competition experiment vs. the original strains. Secondary competition with aged strains was performed as described above, and lasted for 4 days.

### Supercoiling Assay

We adapted a reporter assay to estimate the ability to maintain supercoiling levels in mutants compared to parent strains by inserting a Tn7 cassette containing the *lux* operon under the control of a supercoiling-sensitive *P. aeruginosa* promoter from the gene *PA0614* (Moir et al., 2007). The cassette was inserted as described above. Luminescent strains were grown to mid-exponential phase, then diluted 1:4 in LB+ levofloxacin at 1/4, 1/2, and 1x the measured MIC, and grown in triplicate in a deep well 96-well plate for 7 h. Luminescence was measured using the Envision Multi-Label plate reader (Perkin-Elmer) as well as OD_600_ as described above. Relative Luminescence Units (RLU) was normalized to OD_600_ for all comparisons.

### Mutation Frequency Assay

Mutation frequency was estimated during competition experiments using the spontaneous rifampicin resistance method (Oliver et al., 2000). At each 24-h interval, competition cultures were plated on PIA+ rifampicin at 5x the MIC (ranging from 40 to 500 μg/ml depending on the strain) in addition to plating on PIA. The mutation frequency reported is the number of colonies that grew on PIA + rifampicin after 48 h divided by the number of colonies on PIA alone. Colonies of each strain were differentiated and counted based on fluorescence as described above. For strain 139, 5x the MIC of rifampicin corresponds to 500 μg/ml, which turned the plates a dark shade of red and prevented the differentiation of the fluorescence of the colonies. Thus, for this strain, each rifampicin-containing plate was replica-plated using sterile velvets onto a fresh PIA plate and incubated for an additional 24 h before colonies were counted.

### Statistical Analysis

GraphPad Prism v.6 (GraphPad.com, San Diego, CA, USA) was used to perform statistical analysis. ANOVA was used to compare continuous variables where appropriate, and student’s *t*-test was used to compare mean ratios to the null hypothesis of 1 (indicating no difference). A *p* value ≤ 0.05 denotes significance.

## Results

We previously created isogenic mutants from 6 clinical isolates of *P. aeruginosa* (three *exoU* and three *exoS*) by inserting a point mutation commonly observed in fluoroquinolone-resistant isolates into the *parC* gene (Agnello and Wong-Beringer, 2012). The goal of the current study was to investigate the biological effects of this target site mutation in a controlled genetic background. Mutants are denoted PC^∗^. Interestingly, the addition of the *parC* mutation increased the levofloxacin MIC in only two of the six isolates. Characteristics of mutant and parent strains used in this study are summarized in **Table 1**.

### Growth Rate in Monoculture

As an initial comparison of the relative fitness of mutant vs. parent strains, we tested the doubling time in independent cultures by measuring optical density every 30 min over an 8-h period; results are an average of at least three independent experiments (**Table 1**). Doubling time during exponential phase was calculated by dividing the time interval over the number of generations. Growth rates of all six mutants were comparable to parent strains.

### *parC* Mutation Differentially Affects Competitive Fitness of *exoU* vs. *exoS* Strains

To investigate the fitness costs of the *parC* mutation, we performed head-to-head competition assays. Each PC^∗^ mutant was directly competed against its isogenic parent strain by growth in co-culture for 4 days. Because the strains competed are identical save for the point mutation, any fitness cost is attributable to the mutation. In each individual competition experiment, one strain is tagged with CFP (cyan fluorescent protein), and the other with YFP (yellow fluorescent protein). This allows for the direct enumeration of the number of colonies of each strain after serial dilution onto agar plates. Colonies were counted every 24 h, and the relative fitness of the mutant is determined from the ratio of mutant-to-parent CFU/ml. Each experiment was independently repeated at least five times. We confirmed the neutrality of the YFP and CFP tags by repeating each experiment with each strain carrying the opposite tag. No difference was found, and therefore results from all experiments were combined.

**FIGURE 1:**
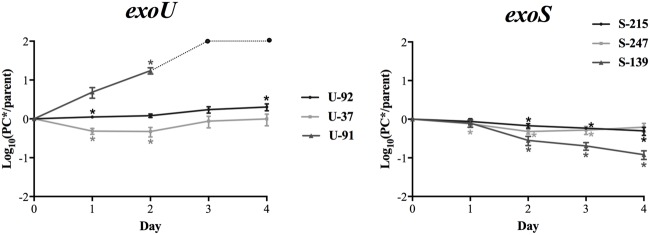
***In vitro* fitness of *exoU* and *exoS*-PC^∗^ mutants.** The average log_10_ CFU/ml ratio of PC^∗^ vs. parent strains per day of *in vitro* competition are shown. Ratios above zero indicate the mutant is more fit, while ratios below zero indicate a fitness cost. The ratio for *exoS* strains generally decreases over the course of the experiment, while the ratios for *exoU* strains either increases or remains stable. Results are an average of at least five independent experiments. Error bars = SEM. For strain U-91, the dotted line and circles indicate ratios after day 2 are beyond the limit of detection. ^∗^Indicates the PC^∗^/parent CFU ratio is significantly different than 1 at *p* < 0.05.

**Figure 1** shows the average mutant-to-parent ratios over time for each strain. Each strain has a unique pattern of fitness costs associated with this mutation; however, in general, there appears to be a higher fitness cost for the *exoS* strains. The *exoS* strains S-139PC^∗^, S-247PC^∗^, and S-215PC^∗^ show a consistent fitness defect over all time points, with strain S-139PC^∗^ the most affected. On the other hand, strain U-92 has the smallest fitness effect, with the mutant/parent ratios ranging from 1.2 to 2.0 over 2–4 days of competition. Strain U-37 shows a similar pattern except the mutant starts with lower fitness (ratio 0.5 at day 2), but catches up to the parent strain by day 4 with a ratio of 0.99. Strain U-91 is unique and interestingly shows a striking increase in fitness due to the mutation; the mutant rapidly outcompetes the parent strain and completely takes over the culture by day 3.

### *exoU*-PC^∗^ Mutants can Better Maintain Wild-Type Supercoiling Levels than *exoS*-PC^∗^ Mutants

**FIGURE 2:**
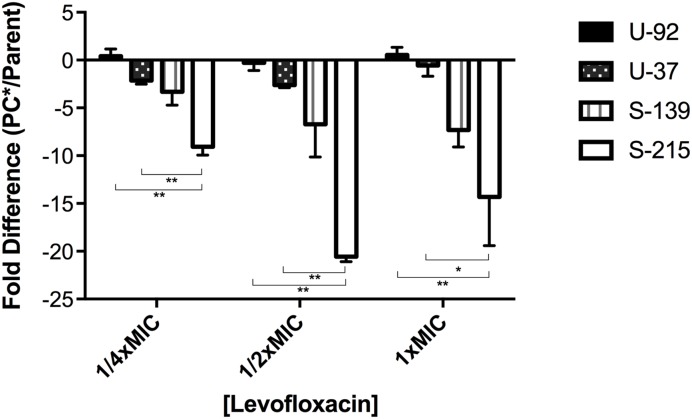
**Supercoiling changes in PC^∗^ mutants compared to parent strains.** Each strain contains a chromosomal reporter construct in which the *lux* operon is under the control of a supercoiling-sensitive promoter. Strains were grown in concentrations of levofloxacin equal to 1/4, 1/2, and 1x the MIC. Luminescence was normalized to cell count using OD_600_. Results are an average of three independent experiments, and error bars represent SEM. ^∗^*p* < 0.05; ^∗∗^*p* < 0.01.

We assessed supercoiling of parent vs. mutants in *exoU* strains U-92 and U-37 and *exoS* strains S-215 and S-139 by inserting a Tn7 genetic element in which a supercoiling sensitive promoter controls the *lux* operon (Moir et al., 2007). Due to challenges inserting the reporter, only two strains of each background were chosen as representative strains. Strains with the insertion were grown under levels of levofloxacin at and below the MIC in order to maximally induce expression of the reporter, and luminescence values were normalized to OD_600_. We measured the level of luminescence in the parent strain as an indirect reporter of the baseline level of supercoiling; any difference in luminescence expression for the PC^∗^ strain reflects a change in supercoiling due to the *parC* mutation. Results show that the PC^∗^ mutants in general have decreased luminescence compared to parent strains; however, this decrease was significantly more pronounced in *exoS*-PC^⋆∗^ strains (**Figure 2**), suggesting that *exoS* strains with the PC^∗^ mutation are somehow less able to maintain the supercoiling level of the parent strains, which may have negative effects on fitness. This is surprising given that the primary role of topoisomerase IV (ParC) is decatenation of daughter replicons and not regulation of supercoiling (Hooper, 2001); however, in this context it may suggest an overall difference in supercoiling regulation in *exoU* vs. *exoS* strains.

### Analysis of Aged Strains

Because, we observed that the fitness of *exoU*-PC^∗^ mutants tended to increase after 24 h of competition, we investigated whether stable changes were occurring during competition that allowed the strains to compensate for fitness costs associated with the *parC* mutation. We collected colonies of parent and PC^∗^ strains from a primary competition experiment (parent vs. mutant) after 7 days and labeled these ‘aged’ strains. In addition, we investigated the effect of sub-inhibitory levels of fluoroquinolone exposure on competitive fitness and ability to compensate for fitness costs by collecting aged strains from primary competition experiments in which 1/8 MIC of levofloxacin was added to the media.

We were not able to collect aged colonies from the primary competition experiments of strain 91 due to the vast difference in fitness between the PC^∗^ mutant and parent strains; the PC^∗^ strain completely overtook the culture by day 3. Because this left us with only two *exoU* strains (U-92 and U-37), we chose two *exoS* strains in order to have an equal number in each group for comparison. We chose strains 215 and 139 because these represent both ends of the spectrum in terms of fitness of the PC^∗^ mutants *in vitro*; strain S-215PC^∗^ shows the least fitness cost of all the *exoS* strains, while strain S-139PC^∗^ exhibits the greatest fitness cost.

Sequencing the FQ-target site genes of the aged strains revealed that no changes occurred in the genes associated with FQ-resistance except for *exoU* strain U-92, in which the parent acquired the Ser87Leu substitution in ParC during competition under levofloxacin exposure (+LVX) at 1/8 MIC. All PC^∗^ strains maintained the *parC* mutation. The MIC to levofloxacin did not change in any of the aged strains (data not shown).

### Aged PC^∗^ Mutant *exoU* vs. *exoS* Strains Show Dramatic Difference in Fitness

**FIGURE 3:**
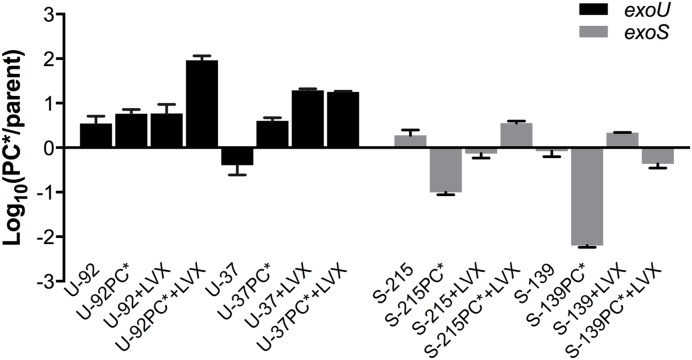
**Fitness of aged strains.** Strains were collected at the end of a 7-day primary competition experiment (PC^∗^ vs. parent) with and without the addition of sub-inhibitory concentration of levofloxacin (+LVX) and subsequently re-competed in a secondary competition experiment (aged vs. un-aged) with or without levofloxacin at the same concentration. Results shown reflect the average fitness of aged strains after 48 h of competition. Experiments were performed in duplicate. Error bars = SEM.

To investigate whether the aged strains had acquired adaptations during competition that rendered them more fit, we compared the fitness of each aged strain to its un-aged counterpart. Aged strains were subjected to a secondary competition experiment in which they were competed against the original, un-aged strain that contained the opposite fluorescent tag, enabling the discrimination and enumeration of each strain during competition. Results reflect the aged-to-un-aged ratio at Day 2 (**Figure 3**). Because each aged strain was competed directly against an un-aged version of itself, we were able to directly observe if fitness had changed in the aged strains. All parent strains had a negligible change in fitness. Both *exoU*-PC^∗^ aged strains increased in fitness greater than fourfold, while the *exoS*-PC^∗^ aged strains showed a 50% decrease in fitness; S-139PC^∗^ was greater than 100 times less fit than before aging.

We also investigated whether growth under sub-inhibitory levels of levofloxacin affected fitness, and if mechanisms to compensate for fitness costs evolved under these conditions. The addition of levofloxacin did not affect the results of primary competition experiments (data not shown), but notable results were seen in the secondary competition experiments of the aged vs. un-aged strains, which also included 1/8 MIC of levofloxacin. Upon subsequent exposure to the drug, *exoU*-PC^∗^ strains were much more fit, outcompeting the un-aged strain rapidly. Specifically, the aged PC^∗^ mutants of *exoU* strains U-37 and U-92 outcompeted the un-aged strains significantly and rapidly, much more so than in the competition experiments without the drug. Interestingly, the measured MICs of the aged and un-aged strains remain unchanged. *exoS*-PC^∗^ strains showed conflicting results; S-215PC^∗^ had gained fitness, while S-139PC^∗^ had slightly decreased fitness (**Figure 3**).

### Mutation Frequency

**FIGURE 4:**
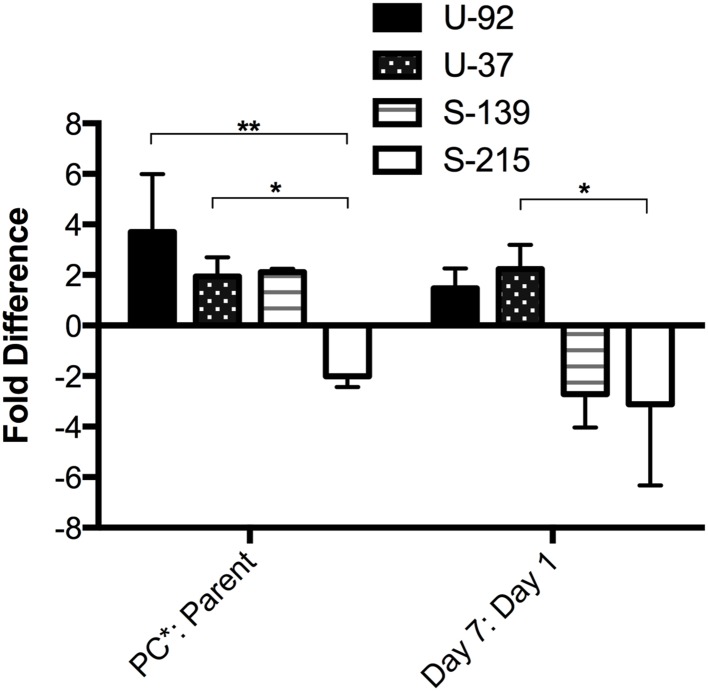
**Mutation frequency of PC^∗^ mutants vs. parent strains measured during competition experiments.** The frequency of spontaneous rifampicin resistance was used to estimate mutation frequency at each time point during a 7-day competition experiment. Bars represent fold difference of the PC^∗^ vs. parent at Day 7 and the fold difference at Day 7 vs. Day 1 for each PC^∗^ strain. Error bars = SEM. ^∗^*p* < 0.05; ^∗∗^*p* < 0.01.

Compensation for potential fitness costs of a resistance-conferring mutation can evolve through the accumulation of beneficial mutations. Therefore, we investigated whether the rate of spontaneous mutation frequency could explain the increase in fitness during competition that was seen in *exoU*-PC^∗^ strains. Mutation frequency was estimated by calculating the frequency of spontaneous resistance to rifampicin (Rif^R^) (Oliver et al., 2000). We estimated mutation frequency during the primary competition experiments for *exoU* strains U-92 and U-37, and *exoS* strains S-215 and S-139 by plating the mixed competition culture on agar containing 5x the MIC of rifampicin at each time point (Days 1–7), and counting the number of colonies based on fluorescence of each strain that were able to grow on the rifampicin-containing plates compared to the overall CFU counts. At Day 7, the *exoU*-PC^∗^ strains have both a higher mutation frequency than the parent strains as well as a higher mutation frequency compared to Day 1. *exoS* strain S-215PC^∗^ had a lower frequency at Day 7 compared to its parent strain as well as a lower frequency compared to Day 1. Surprisingly, *exoS* strain S-139PC^∗^, which is significantly less fit than its parent strain, has a twofold higher mutation frequency than the parent strain at Day 7. However, its mutation frequency at Day 7 decreased almost threefold from Day 1 (**Figure 4**). Therefore, mutation frequencies seem to generally correlate with fitness results.

## Discussion

*Pseudomonas aeruginosa* is considered one of six bacteria that pose an immediate threat to public health, according to the Infectious Diseases Society of America, due to its increasing prevalence, broad arsenal of virulence factors, and emergence of resistance to all available antibiotics (Boucher et al., 2009). In its recent report entitled “Antibiotic Resistance Threats in the United States” the Centers for Disease Control named *P. aeruginosa* a serious threat that requires prompt action and continual monitoring to prevent a worsening of the problem of resistance (Centers for Disease Control [CDC], 2013). Notably, resistance rates of *P. aeruginosa* to the once-effective fluoroquinolone (FQ) antibiotics are now greater than 30% in the United States, with many of those strains also multi-drug resistant (Rosenthal et al., 2012).

During acute disease, *P. aeruginosa* utilizes the toxins of the type III secretion system to circumvent the host immune system and establish infection. Of the four exotoxins, *exoU* is the most virulent, encoding a potent phospholipase that disrupts the plasma membrane and leads to rapid cell death (Sato et al., 2003). ExoU activity induces excessive inflammation and tissue damage in the host as well as increased bacterial dissemination that can lead to septic shock (Kurahashi et al., 1999) and higher rates of mortality compared to infections with ExoS-secreting strains (Shaver and Hauser, 2004; Zhang et al., 2014; Peña et al., 2015).

As the problem of antibiotic resistance continues to worsen, it is becoming apparent that the evolution of virulence factors and antibiotic resistance cannot be considered strictly independent processes (Beceiro et al., 2013; Hauser, 2014). Previously, we found that infection by FQ-resistant *P. aeruginosa* was an independent predictor for threefold higher mortality or prolonged illness by an additional 5 days compared to those infected with FQ-susceptible strains (Hsu et al., 2005). In a separate study, we reported that among patients from whom *P. aeruginosa* was isolated from the respiratory tract, strains with the combined traits of *exoU* and FQ-resistance were much more likely to cause pneumonia than *exoS*, FQ-resistant strains or either trait alone (Sullivan et al., 2014).

Analysis of 270 of our *P. aeruginosa* clinical isolates led to the observation that *exoU* strains are not only enriched in the FQ resistant sub-population, but are also more likely to have acquired two or more target site mutations than *exoS* strains, especially in the *parC* gene (Agnello and Wong-Beringer, 2012). In order to study the biological effects of a resistance-conferring mutation in a controlled, isogenic background, we adapted a technique for inserting point mutations into bacterial genomes for use in our clinical isolates (Swingle et al., 2010). Using the technique of oligonucleotide recombination, we created 6 FQ-resistant mutants (three *exoU* and three *exoS*) (Agnello and Wong-Beringer, 2014) from clinical strains by inserting a mutation into the *parC* gene.

Because the target site mutation, we inserted in *parC* rarely occurs in clinical isolates without a pre-existing mutation in *gyrA* (Jalal and Wretlind, 1998), we chose clinical strains that had a pre-existing *gyrA* mutation (Thr83Ile) in order to mimic FQ-resistant strains encountered in the clinical setting. These specific point mutations in *gyrA* and *parC* are commonly found in FQ-resistant clinical strains of *P. aeruginosa* (Nakano et al., 1997; Mouneimné et al., 1999; Higgins et al., 2003; Agnello and Wong-Beringer, 2012). The goal of the current study was to assess the biological effects on fitness of the *parC* mutation and determine if the magnitude of the effects differ in *exoU* vs. *exoS* strains.

The acquisition of resistance is generally thought to be accompanied by a fitness cost to the bacterium (Andersson and Levin, 1999); however, there are many reports of neutral fitness effects or enhanced fitness due to resistance-conferring mutations. Evidence from FQ-resistant *E. coli*, *S. pneumoniae, and N. gonorrhoeae* suggest that a secondary resistance mutation does not further decrease fitness and may restore the low fitness of primary mutants back to wild-type levels (Komp Lindgren et al., 2005; Rozen et al., 2007; Marcusson et al., 2009; Kunz et al., 2012; Machuca et al., 2014). Furthermore, many fitness effects seem to be strain dependent, as was shown in a study of ciprofloxacin-resistant *Campylobacter jejuni*, in which a *gyrA* mutation conferred a high cost to one strain but actually increased fitness for a different strain (Luo et al., 2005). Similar results were seen in *S. pneumoniae*, in which some FQ-resistant mutant strains had increased fitness while others had a significant cost (Balsalobre and de la Campa, 2008). This suggests that fitness effects are highly dependent on strain genetic background, which may explain the differences, we have seen between *exoU* and *exoS* strains.

Head-to-head competition assays are a standard method for investigating the relative fitness of a mutant strain compared to its isogenic parent strain, and it is possible to detect differences in fitness as small as 1% (Andersson and Levin, 1999; Corzett et al., 2013). Strains are mixed together in co-culture, and must compete for the limited available resources (Finkel, 2006; Kraigsley and Finkel, 2009). In most studies, strains are differentiated based on selective growth on antibiotic plates. However, since the *parC* mutation, we inserted did not increase the levofloxacin MIC in all strains, we had to develop a different approach. We took advantage of a Tn7-based system developed by Schweizer (Choi et al., 2005) in order to insert a cassette containing either YFP or CFP under the control of a strong promoter.

The growth of the PC^∗^ strains did not differ dramatically from those of the parents when grown in monoculture; however, differences were seen when strains had to compete for resources in the same culture flask during competition experiments. Overall, the *parC* mutation imposed a moderate to considerable fitness cost on *exoS* strains, while *exoU* strains were able to better tolerate the mutation. Notably, fitness of *exoU*-PC^∗^ strains ranged from maintaining the wild-type level of fitness to outcompeting the parent strain by more than 10-fold, whereas *exoS*-PC^∗^ strains were consistently less fit than parent strains.

The FQ target site mutation investigated in the current study occurs in a topoisomerase enzyme and therefore may have an effect on the regulation of supercoiling. Common methods for investigating supercoiling levels rely on reporter plasmids that need to be selected for and maintained. Because, we are using clinical isolates, there is a high level of multi-drug resistance that precludes the use of standard selection antibiotics. To circumvent this, we inserted a Tn7 element (Moir et al., 2007) onto the chromosome in which a supercoiling sensitive promoter controls *lux* expression. *exoU*-PC^∗^ mutants were better able to maintain the supercoiling levels of the parent strains, while *exoS*-PC^∗^ mutants showed a more drastic change in supercoiling, as observed by decreased *lux* expression. Other studies have shown that changes in supercoiling are correlated with changes in fitness (Kugelberg et al., 2005; Marcusson et al., 2009), and therefore the regulation of supercoiling may reflect the level of overall fitness and may explain the fitness differences seen in our strains. The ability of *exoU*-PC^∗^ strains to better regulate supercoiling levels under the stress of FQ exposure reflects increased fitness of these strains overall. The supercoiling level of the cell affects global gene expression and replication efficiency, and changes in supercoiling may alter response to environmental stressors, or even modulate pathogenesis in the host (Redgrave et al., 2014).

In the primary competition experiments, *exoU*-PC^∗^ mutants tended to increase in fitness over the course of the 4-day experiment. This suggests that *exoU* strains may be acquiring beneficial adaptations that allow for the compensation of the fitness costs associated with the PC^∗^ mutation. Bacteria have the ability to rapidly evolve compensatory mechanisms to mitigate the fitness costs associated with antibiotic resistance, and compensatory mechanisms can reverse fitness costs without any loss of resistance (Andersson and Hughes, 2010). The *exoU*-PC^∗^ strains that had been ‘aged’ through a primary competition experiment for 7 days have increased fitness compared to the un-aged strains, while aged *exoS*-PC^∗^ strains have decreased fitness. The results suggest that the compensatory mechanisms in *exoU* strains most likely are acting as a repressor of the negative fitness effects of the *parC* mutation, as opposed to just conferring a general gain in fitness, based on the observation that the aged parent strains did not show as much of an increase in fitness as the PC^∗^ mutants. The observed ability of *exoU* strains to compensate for the costs of FQ-resistance has many clinically negative consequences. Compensation in clinical populations leads to the stabilization of resistant populations without the presence of drug (Andersson and Hughes, 2010). Increased fitness after acquisition of FQ-resistance adds to the already complicated problem of treating infections caused by highly virulent *exoU* strains.

The inability of *exoS*-PC^∗^ strains to regulate supercoiling suggests that the compensatory mechanisms in *exoU* strains are acting to maintain wild-type supercoiling levels in the PC^∗^ mutants, as has been reported in other studies of compensation of the costs of FQ-resistance (Kugelberg et al., 2005; Marcusson et al., 2009). Although, we did not identify the exact mechanisms responsible for the compensatory effects, we have shown that they are stable since strains were frozen and grown before testing for compensation in secondary competition experiments, and the results were reproducible in repeated experiments. Sequencing of the quinolone-resistance determining regions of *gyrA/B* and *parC/E* revealed no additional mutations had occurred during competition; however, beneficial mutations may have occurred elsewhere. For both *exoU* strains tested, the mutation frequencies correlated with the increase in fitness observed during competition experiments, suggesting that higher mutation frequency may facilitate the acquisition of beneficial mutations that mitigate the fitness costs of the *parC* mutation.

A dramatic increase in fitness occurred in the aged *exoU*-PC^∗^ strains taken from a primary competition experiment in which sub-inhibitory levels (1/8 the MIC) of levofloxacin were added to culture. Although this low level of drug did not affect the growth of strains in the primary competition experiment, when the aged *exoU*-PC^∗^ strains were re-competed under the same concentration of levofloxacin, they were 20–100 times more fit than before aging. In contrast, *exoS*-PC^∗^ strains were dramatically less fit under these circumstances. However, none of the aged PC^∗^ strains had an increase in MIC, nor any additional FQ-resistance mutations in target site genes. These results suggest that although the level of levofloxacin was much below the MIC, highly fit strains were selected for rapidly, more so than in conditions without drug. Also, the highly fit strains were not more resistant, suggesting that perhaps the presence of levofloxacin accelerated the process of compensation for the already present resistance mutations. As sub-inhibitory concentrations of antibiotics are routinely present during treatment due to insufficient dosing or inadequate penetration to certain areas of the body (Baquero and Negri, 1997; Andersson and Hughes, 2014), the implications of our results are alarming and suggest that low levels of antibiotic can rapidly select for highly fit strains, preferentially the highly virulent *exoU*-containing strains. Furthermore, when these highly fit, highly virulent strains are re-introduced to the antibiotic, they will rapidly outcompete all other strains. The sub-inhibitory concentration was also able to select for a *parC* mutation in an *exoU* parent strain, emphasizing the known phenomenon that selection for resistance mutations can occur at sub-inhibitory levels of antibiotic (Baquero et al., 1998; Andersson and Levin, 1999; Gullberg et al., 2011; Andersson and Hughes, 2014).

Overall, these results suggest a potentially lower fitness burden associated with FQ-resistance for *exoU* strains than for *exoS* strains, which in part provides an initial biological explanation for *exoU* strains’ greater tendency to acquire FQ-resistance in the clinical setting. Although, we have yet to identify the specific genetic components underlying the fitness differences, we suspect that genes unique to strains with the *exoU* genetic background may contribute to the fitness benefits seen in this study. The *P. aeruginosa* genome consists of a highly conserved core genome, but variability is introduced in the form of genomic islands, which make up the accessory genome. Genes within the islands usually encode accessory activities such as specific pathogenicity or symbiosis factors (Harrison et al., 2010). The *exoU* gene, along with its chaperone *spcU*, is located on a pathogenicity island, a specialized genomic island. *exoU* has been identified as part of a few different pathogenicity islands. The most highly studied is from the reference strain PA14, in which the *exoU* gene is on an island named PAPI-2 (Kulasekara et al., 2006). Studies with PA14 have shown that virulence is dependent on the presence of the entire island and not just the *exoU* gene alone; therefore, other as yet unknown genes contained on the pathogenicity islands contribute combinatorially to the increased virulence of *exoU* strains (Harrison et al., 2010). Furthermore, pathogenicity in strain PA14 requires the coordinated action of multiple virulence factors, associated with both the core and accessory genomes (Lee et al., 2006). Therefore, it is possible that other genes in the accessory genome in combination with *exoU* may provide fitness benefits to *exoU* strains that allow for increased ability to adapt to the fitness costs of FQ-resistance. Genomic islands have been shown to confer fitness benefits, and the accessory genome of *P. aeruginosa* is an important driver of the ability of strains to persist in a particular environment (Hacker and Carniel, 2001; Ozer et al., 2014).

This study has several limitations. We acknowledge that a limited number of clinical strains and their isogenic mutants were evaluated in the study. Despite having access to a large collection of clinical strains, the selection of strains to create isogenic target site mutants to study the effect of FQ-resistance on fitness proved challenging, as most of the isolates in our collection were found to already contain the resistance-conferring mutation that, we are investigating, due to the high prevalence of FQ resistance in clinical isolates of *P. aeruginosa.* Therefore, we were not able to use those to create isogenic pairs. Furthermore, genetic manipulation of these clinical isolates proved to be difficult, as these strains were not universally amenable to the creation of isogenic mutants via oligonucleotide recombination. Nonetheless, it is worth nothing that the strains evaluated in this study were carefully selected to represent a broad range of characteristics such as varying degrees of FQ resistance, virulence, and clinical outcomes. While the limited number of strains evaluated in this study may not necessarily represent the genetic variability present among all *exoU* and *exoS* clinical strains, strain-specific differences in fitness effects were observed even among strains with the same *exoU* or *exoS* genetic background, though the trend observed appears to follow similar pattern for strains with the same *exoU* or *exoS* genetic background.

Understanding the fitness costs of antibiotic resistance and possibilities of compensation for these costs is essential for rationally combating the problem of antibiotic resistance. Importantly, this study outlines a useful model with the creation of isogenic clinical strains for investigating the biological costs of resistance in a medically important pathogen and can be applied to other drug-organism pairs.

## Author Contributions

MA, AW-B, and SF designed the experiments. MA and AW-B wrote the manuscript. SF edited and provided critical review of the manuscript.

## Conflict of Interest Statement

The authors declare that the research was conducted in the absence of any commercial or financial relationships that could be construed as a potential conflict of interest.
